# DIVERSITY OF DAILY EXPERIENCES: SOCIOECONOMIC AND PSYCHOLOGICAL CORRELATES AND IMPLICATIONS FOR HEALTHY AGING

**DOI:** 10.1093/geroni/igae098.0343

**Published:** 2024-12-31

**Authors:** Soomi Lee, Rachel Koffer, David Almeida

**Affiliations:** Chair; Co-Chair; Discussant

## Abstract

As intensive longitudinal data collection becomes more widespread, innovative methods to quantify daily and momentary experiences are needed to advance theoretical understanding of daily life and its implications for successful aging. A particularly promising area of advancement is in quantitative summaries of rich categorical data regarding daily experiences (e.g., types of activities including physical activity, chores, volunteering, etc.; types of stressors including work stressors, arguments, social network stressors, etc.; types of social engagements, including with church groups, neighbors, family, etc.). This symposium will discuss a framework for understanding antecedents and outcomes of diversity of daily experiences and provide exemplary studies within this framework. First, Koffer will present a new X-versity theoretical perspective, an innovative approach to understanding the richness and balance inherent in diverse daily experiences and its significance in the context of healthy aging. Three domains of daily experiences (activities, stressors, and social interactions) will then be represented in empirical presentations to discuss socioeconomic and psychological correlates of diversity of daily experiences. Santos finds higher income relates to higher activity diversity between 2011-2022, with differences by race/ethnicity. Surachman finds more financial hardship relates to higher stressor concentration (narrow and unbalanced stressor experiences in daily life), particularly for older adults. Jeon finds COVID-19 pandemic-driven decreases in social activity diversity related to increased loneliness. The Discussant, Almeida will provide an overview of the advancements in daily diary and intensive longitudinal study of everyday life and discuss implications of diversity of daily experiences for health and well-being across the adult life span. Health Behavior Change Interest Group Sponsored Symposium

